# Common Evaluations of Disease Activity in Rheumatoid Arthritis Reach Discordant Classifications across Different Populations

**DOI:** 10.3389/fmed.2018.00040

**Published:** 2018-03-08

**Authors:** Helena Canhão, Ana Maria Rodrigues, Maria João Gregório, Sara S. Dias, José António Melo Gomes, Maria José Santos, Augusto Faustino, José António Costa, Cornelia Allaart, Emilia Gvozdenović, Desirée van der Heijde, Pedro Machado, Jaime C. Branco, João Eurico Fonseca, José António Silva

**Affiliations:** ^1^CEDOC, EpiDoC Unit, Nova Medical School, Nova University, Lisbon, Portugal; ^2^National School of Public Health, Nova University, Lisbon, Portugal; ^3^Instituto Português de Reumatologia, Lisbon, Portugal; ^4^Rheumatology Research Unit, Instituto de Medicina Molecular, Lisbon Academic Medical Center, Lisbon, Portugal; ^5^Rheumatology Department, Hospital Garcia de Orta, Almada, Portugal; ^6^Centro Hospitalar Alto Minho, Ponte de Lima, Portugal; ^7^Rheumatology Department, Leiden University Medical Center, Leiden, Netherlands; ^8^Centre for Rheumatology Research, MRC Centre for Neuromuscular Diseases, University College London, London, United Kingdom; ^9^Rheumatology Department, Centro Hospitalar Lisboa Ocidental – HEM, Lisbon, Portugal; ^10^Rheumatology Department, CHLN – Santa Maria Hospital, Lisbon Academic Medical Center, Lisbon, Portugal; ^11^Faculdade de Medicina da Universidade de Coimbra, Centro Hospitalar Universitário de Coimbra, Coimbra, Portugal

**Keywords:** rheumatoid arthritis, disease activity, patient reported outcomes, physicians’ perspective, acute phase reactants, DAS28-ESR, METEOR

## Abstract

**Objectives:**

The classification of disease activity states in rheumatoid arthritis (RA) can be achieved through disease activity indices, such as the Disease Activity Score in 28 joints erythrocyte sedimentation rate (DAS28-ESR), the Simplified Disease Activity Index (SDAI), and the Clinical Disease Activity Index (CDAI). Subjective measurements, such as patient reported outcomes have been incorporated into several of these indices alongside more objective assessments, such as increases in the ESR and C-reactive protein. Moreover, while they use similar criteria, different indices weight these criteria to different extents. Therefore, the classifications based on each evaluation may not always be the same. We aim to compare the performance of the three indices and their individual components in two different populations.

**Methods:**

Data from Dutch and Portuguese adherent centers were extracted from the METEOR database, a multinational collaboration on RA. We included a total of 24,605 visits from Dutch centers (from 5,870 patients) and 20,120 visits from Portuguese centers (from 3,185 patients). We compared the disease activity states as evaluated by the DAS28-ESR, CDAI, and SDAI across the two populations. In addition, we analyzed the individual components of each evaluation, including their respective contributions to the outcome, in each population.

**Results:**

We found significant differences in the disease activity states classified with the DAS28-ESR between the two populations. SDAI and CDAI had more congruous results. While the proportion of visits to Dutch and Portuguese centers that were classified as “in remission” was very similar between the CDAI and SDAI, the DAS28-ESR gave discordant results. Dutch patients had lower ESRs, which is more heavily weighted in the DAS28-ESR. In addition, even though the mean physicians’ global assessment values did not vary significantly for Dutch vs Portuguese physicians, we found that doctors at Portuguese centers overall scored the physician’s global assessment lower than Dutch physicians for patient visits classified by disease activity state.

**Conclusion:**

While the CDAI and SDAI assigned disease activity states that were largely similar, the DAS28-ESR was often discordant across the two populations. Moreover, we found that physicians, more than patients, evaluated disease activity differently among the Portuguese and Dutch populations.

## Introduction

Rheumatoid arthritis (RA) is a chronic, inflammatory disease that can affect joints, resulting in pain and discomfort. Patient reported outcomes (PROs) are increasingly being included in clinical trials and clinical practice to evaluate pain, function, and quality of life. PROs have also been incorporated into several of the major disease activity indices alongside more objective assessments, such as increases in the erythrocyte sedimentation rate (ESR) and C-reactive protein (CRP). Specifically, the Disease Activity Score in 28 joints (DAS28-ESR) (1), the Simplified Disease Activity Index (SDAI) (2), and the Clinical Disease Activity Index (CDAI) (3) are the disease activity indices that are most frequently used in RA, which generate specific cut-off values that are used to classify RA as in remission or in a low, moderate, or high activity state (4–6).

DAS28-ESR (1) is the most commonly used evaluation in daily practice, as well as in clinical trials, where it has also been validated for assessment of treatment response (4, 7, 8). In contrast, SDAI (2) and CDAI (3) were developed later than DAS28-ESR (5), although the SDAI is included in the American College of Rheumatology (ACR)/European League Against Rheumatism (EULAR) remission criteria (9) and more often used in clinical trials. All three evaluations rely on composite scores and include similar individual components; however, the components are weighed differently in each assessment (3). In addition, the evaluations incorporate both objective and subjective components, including both patient and physician perspectives, which are not always the same. As such, there is the potential for variability between these indices, and our previous work, along with that of others, has reported disagreements (10, 11). This could be concerning, as physicians use the scores obtained to make decisions regarding proper treatment and dosing, although they also take into account the patient’s clinical history and perspectives, as well as their own professional observations.

Therefore, the primary objective of this study was to compare the performance of the three indices and their individual components in two different populations. Since patient and physician perspectives can vary based on factors such as education and culture, we compared these different measures in two distinct populations, using the Measurement of Efficacy of Treatment in the Era of Rheumatology (METEOR) multinational database.

## Materials and Methods

### Patients and Visits

Data from Dutch and Portuguese adherent centers were extracted from the METEOR database, a multinational collaboration on RA, from 2008 until 2013 (12). The database provides data on patient- and physician-reported outcome measures in RA. De-identified data have been longitudinally collected in the central database. Data collection from both countries started in 2008 (12, 13). Currently, the tool is used worldwide and includes more than 50,000 patients. Data from Dutch centers were directly inserted in METEOR, by physicians and clinical nurses. Netherlands data included patients from one university hospital (the majority) and several other centers of secondary care from the western part of the Netherlands. Conversely, Portuguese visits were initially registered in Reuma.pt, the nationwide Portuguese Rheumatic Diseases Register, from the Portuguese Society of Rheumatology that comprises contribution from 90% of Portuguese rheumatology centers (academic centers, public, and private hospitals) (13), and then were posteriorly exported to METEOR. Data acquisition was made by rheumatologist or rheumatology nurses. All RA patients fulfilled the 1987 ACR criteria for the diagnosis of RA (14). Our data set contained information from 9,055 RA patients for a total of 44,725 visits (24,605 visits from 5,870 Dutch patients and 20,120 visits from 3,185 Portuguese patients). Reuma.pt was approved by the Portuguese National Data Protection Board and the ethics committees of the participating hospitals. Patients provided written informed consent for registry participation. METEOR was approved by the local ethics committees and is adherent with European General Data Protection Regulation guidelines. All study procedures were in accordance with the Declaration of Helsinki. The METEOR tool used only de-identified data and all personal information was encrypted locally.

### Measurements

Information was obtained about tender joint count based on 28-joint assessment (TJC28), swollen joint count based on 28-joint assessment (SJC28), physician’s global assessment of RA disease activity on a 100 mm visual analog scale (MDGA), patient’s global assessment of activity on a 100 mm analog scale, ESR and CRP (mg/l). Patients’ characteristics and number of visits were also collected (gender, age, disease duration, and disease diagnosis). The three clinical activity disease indices, the DAS28-ESR, SDAI, and CDAI were calculated as previously described (1–3).

### Disease Activity Definitions

The three disease activity indices (DAS28-ESR, CDAI, and SDAI) and their respective validated cut-offs were used to define remission, low disease activity, moderate disease activity, and high disease activity as described in Table 1 (4–7).

**Table 1:** Cutoff values for different disease activity states.

Index	Remission	Low	Moderate	High
DAS28-erythrocyte sedimentation rate	<2.6	≥2.6 and ≤3.2	>3.2 and ≤5.1	>5.1
CDAI	≤2.8	>2.8 and ≤10	>10 and ≤22	>22
SDAI	≤3.3	>3.3 and ≤11	>11 and ≤26	>26

*DAS, disease activity score; CDAI, clinical disease activity index; SDAI, simplified disease activity index*.

For remission definition, the 2011 ACR/EULAR Boolean remission criteria were also applied in this study. In the 2011 ACR/EULAR Boolean remission criteria, all TJC28, SJC28, CRP (mg/dl), and PGAvalues should be ≤1 (9).

### Statistical Analysis

The demographic and clinical characteristics of the two populations were compared using chi-squared tests and *t*-tests, respectively, for discrete and continuous variables. DAS28-ESR, CDAI, and SDAI scores were calculated and at each visit the disease activity state was classified according to previously established cutoffs (4–6). We calculated the relative contribution of the individual components of DAS28-ESR, CDAI, and SDAI scores. Descriptive statistics for the RA core set measures were calculated. *Z*-tests for equality of two independent proportions and for equality of two independent means were used to compare the proportions of visits and the score means, respectively, at each disease activity state for each index and population. Correlation between TJC28, SJC28, ESR, and CRP was done using Pearson correlation test. Missing data were not imputed.

To eliminate the effect of the number of visits per patient, we performed a sensitivity analyses where we analyzed a subset of data containing one visit per patient from each population. For that, we wrote a Visual Basic for Applications (VBA) script that computed the total number of visits for each patient and, for each one of them, generated a random number between one and the total number of visits obtained. Then, for each patient, we selected the visit corresponding to the randomly generated number. In this sample of one random visit per patient, we independently performed the same analyses described above for the group with all visits. All available visits were analyzed using Stata or R programming. Statistical significance was determined when *p*-values were less than 0.05.

## Results

A total of 44,725 rheumatology visits, from 9,055 RA patients, were included in our analysis. Of those, 5,870 patients were seen in Dutch centers, accounting for 24,605 visits, and 3,185 patients were seen in Portuguese centers, accounting for 20,120 visits. Information on the patients and their visits is described in Table 2.

**Table 2:** Characteristics of patients and their visits.

	Dutch		Portuguese		
	*N*	Patient	*N*	Patient	
**A. Patients’ characteristics**					

Patients	5,870	69.5	3,185	82.1	
Female (%)	3,923		2,616		
Age (years) (mean ± SD)	5,489	62.6 ± 14.7	3,180	61.7 ± 14.2	
Disease duration (years) (mean ± SD)	2,278	11.8 ± 8.9	2,305	15.0 ± 10.1	
Diagnosis duration (years) (mean ± SD)	2,520	10.4 ± 8.7	2,397	12.8 ± 9.0	

**B. Visit characteristics**					***p*-Value**

Visits	24,605	35.3 ± 23.5	20,120	38.3 ± 25.4	<0.0001
PGA (mean ± SD)	21,292		14,618		
MDGA (mean ± SD)	8,119	27.4 ± 20.9	10,160	27.1 ± 21.8	0.33
TJC28 (mean ± SD)	22,272	2.5 ± 3.9	17,774	4 ± 5.8	<0.0001
SJC28 (mean ± SD)	22,104	1.6 ± 2.7	17,774	2.4 ± 3.9	<0.0001
ESR (mean ± SD)	20,990	19.1 ± 17.9	16,886	26.7 ± 22.6	<0.0001
C-reactive protein (mean ± SD) (mg/l)	3,724	10.1 ± 18.7	15,539	11.6 ± 21.9	<0.0001

*PGA, patient assessment of disease activity (100 mm); MDGA, physician assessment of disease activity (100 mm); TJC28, 28 tender joint count; SJC28, 28 swollen joint count; ESR, erythrocyte sedimentation rate (mm/h)*.

*Chi-square and t-tests were used, as appropriate. Significance: p-value < 0.05*.

The individual components of the disease activity scores, including acute phase reactants, were significantly higher for the Portuguese visits, with the only exception being the MDGA, which did not differ between groups.

We next performed an analysis of each disease activity category as classified by the DAS28-ESR, the CDAI, and the SDAI. Table 3 presents the proportion of Dutch and Portuguese visits in which the patient was classified into each disease activity category. We found that the proportion of patient visits in each disease category as classified by the DAS28-ESR was significantly different between the two populations. Specifically, significantly more visits to Portuguese centers involved patients classified as moderate or high disease activity by the DAS28-ESR than the Dutch centers. In comparison, the CDAI and SDAI results had a more homogenous distribution across visits. In addition, for the remission category, no significant differences were detected between the two populations according to the CDAI, SDAI, and ACR/EULAR 2011 remission criteria (9).

**Table 3:** The number and percentage of visits in each population according to disease activity state and corresponding *z*-tests for equality of two independent proportions.

	Dutch visits		Portuguese visits			
	*N*	%	*N*	%	*z*	*p*-Value
DAS28-ESR remission	7,713	39.17	3,890	27.77	12.56	<0.0001
DAS28-ESR low	3,583	18.20	2,157	15.40	2.77	0.005
DAS28-ESR moderate	6,783	34.45	5,354	38.22	−4.28	<0.0001
DAS28-ESR high	1,610	8.18	2,608	18.62	−10.20	<0.0001
CDAI remission	1,298	16.93	1,489	14.83	1.51	0.13
CDAI low	3,266	42.59	3,874	38.58	3.44	0.0006
CDAI moderate	2,262	29.50	2,833	28.21	1.00	0.32
CDAI high	843	10.99	1,846	18.38	−5.26	<0.0001
SDAI remission	292	16.88	1,433	15.42	0.61	0.54
SDAI low	543	31.39	3,508	37.75	−2.96	0.003
SDAI moderate	636	36.76	2,890	31.10	2.70	0.007
SDAI high	259	14.97	1,461	15.72	−0.31	0.76
ACR/European League against rheumatism remission	393	13.04	1,622	12.27	0.41	0.68

*N, number of visits; z, z-test for equality of two independent proportions*.

*DAS28-ESR, disease activity score in 28 joints; CDAI, clinical disease activity index; SDAI, simplified disease activity index*.

*z > 0 when the proportion in Dutch group is higher than in Portuguese group*.

We also compared the individual components of the DAS28-ESR, CDAI, and SDAI between the two populations at each disease activity state (Tables 4 and 5). With the exception of the high disease activity category, we found ESR to be the individual parameter that differed the most between the Dutch and Portuguese visits. In addition, even though the mean physician’s global assessment values did not vary significantly for Dutch vs Portuguese physicians, we found that doctors at Portuguese centers overall scored the physician’s global assessment lower than Dutch physicians for patient visits with same disease activity state.

**Table 4:** The mean scores for the individual components of each disease activity evaluation, according to disease activity category.

	Dutch visits				Portuguese visits			
	Remission	Low	Moderate	High	Remission	Low	Moderate	High
**A. DAS28-ESR**								
PGA	20.5 ± 16.7	32.3 ± 18.2	46.3 ± 19.9	68.0 ± 17.1	18.2 ± 18.0	30.0 ± 19.0	43.2 ± 20.7	64.6 ± 19.9
TJC28	0.4 ± 0.8	1.2 ± 1.6	3.5 ± 3.2	10.2 ± 5.7	0.3 ± 0.8	0.9 ± 1.5	3.7 ± 3.5	12.8 ± 6.8
SJC28	0.3 ± 0.9	0.8 ± 1.4	2.1 ± 2.4	6.4 ± 4.5	0.3 ± 0.8	0.7 ± 1.4	2.1 ± 2.6	7.2 ± 5.4
ESR	9.1 ± 7.0	18.9 ± 13.9	25.1 ± 17.9	43.6 ± 25.8	11.2 ± 7.0	22.6 ± 14.6	29.8 ± 20.5	46.6 ± 27.8
DAS28-ESR	1.8 ± 0.6	2.9 ± 0.2	4.0 ± 0.5	5.8 ± 0.6	1.9 ± 0.5	2.9 ± 0.2	4.1 ± 0.5	6.1 ± 0.8

**B. CDAI**								
PGA	6.5 ± 6.0	30.2 ± 14.9	52.7 ± 17.7	68.6 ± 16.6	6.0 ± 6.7	31.6 ± 17.0	50.4 ± 19.0	65.3 ± 19.9
MDGA	5.2 ± 4.7	18.8 ± 10.1	40.2 ± 15.3	61.7 ± 16.2	3.3 ± 4.3	16.0 ± 10.2	35.8 ± 14.8	56.2 ± 18.1
TJC28	0.1 ± 0.3	0.8 ± 1.1	3.4 ± 2.4	11.1 ± 6.0	0.1 ± 0.3	0.9 ± 1.2	4.0 ± 2.8	13.9 ± 6.5
SJC28	0.0 ± 0.2	0.5 ± 0.9	2.2 ± 2.1	6.7 ± 4.9	0.1 ± 0.3	0.6 ± 1.0	2.6 ± 2.3	8.0 ± 5.3
CDAI	1.3 ± 0.9	6.2 ± 2.1	14.9 ± 3.3	30.8 ± 8.2	1.1 ± 0.9	6.3 ± 2.1	15.2 ± 3.4	34.0 ± 10.0

**C. SDAI**								
PGA	5.5 ± 6.2	30.3 ± 15.8	54.5 ± 18.0	68.3 ± 17.8	7.0 ± 7.6	31.6 ± 17.1	50.8 ± 19.3	66.2 ± 19.8
MDGA	4.8 ± 5.0	20.6 ± 11.6	45.9 ± 16.9	65.3 ± 16.1	3.7 ± 4.6	15.8 ± 10.2	36.0 ± 15.0	57.8 ± 17.9
TJC28	0.1 ± 0.4	0.9 ± 1.2	4.3 ± 3.2	13.4 ± 6.9	0.1 ± 0.3	0.9 ± 1.2	4.3 ± 3.3	14.5 ± 6.6
SJC28	0.1 ± 0.2	0.5 ± 0.9	2.0 ± 2.6	7.0 ± 5.8	0.1 ± 0.3	0.6 ± 1.1	2.7 ± 2.4	8.3 ± 5.4
CRP	3.7 ± 3.8	5.8 ± 6.7	9.0 ± 11.4	23.8 ± 37.7	4.1 ± 4.5	6.6 ± 9.2	13.3 ± 19.9	27.9 ± 39.5
SDAI	1.6 ± 1.0	7.1 ± 2.2	17.3 ± 4.1	36.1 ± 9.6	1.7 ± 1.0	7.0 ± 2.2	17.1 ± 4.2	38.0 ± 10.2

*PGA, patient assessment of disease activity (100 mm); MDGA, physician assessment of disease activity (100 mm); TJC28, 28 tender joint count; SJC28, 28 swollen joint count; CRP, C-reactive protein (mg/l); ESR, erythrocyte sedimentation rate (mm/h); DAS28-ESR, disease activity score evaluating 28 joints; CDAI, clinical disease activity index; SDAI, simplified disease activity index*.

*All data are represented as the mean ± SD*.

**Table 5:** Comparison of the mean scores from the individual components of each disease activity evaluation between the Dutch and Portuguese populations.

	Remission		Low		Moderate		High	
	*z*	*p*-Value	*z*	*p*-Value	*z*	*p*-Value	*z*	*p*-Value
**A. DAS28-ESR**								
PGA	6.73	<0.0001	4.49	<0.0001	8.39	<0.0001	5.82	<0.0001
TJC28	6.24	<0.0001	6.84	<0.0001	−2.61	0.0090	−13.61	<0.0001
SJC28	3.65	0.0003	1.94	0.0520	−0.88	0.3791	−5.32	<0.0001
ESR	−14.33	<0.0001	−9.59	<0.0001	−13.41	<0.0001	−3.57	0.0004
DAS28-ESR	−7.34	<0.0001	−0.49	0.6245	−7.94	<0.0001	−10.44	<0.0001

**B. CDAI**								
PGA	1.99	0.0465	−3.71	0.0002	4.64	<0.0001	4.40	<0.0001
MDGA	10.63	<0.0001	11.84	<0.0001	10.4	<0.0001	7.75	<0.0001
TJC28	1.37	0.1713	−3.38	0.0007	−7.95	<0.0001	−11.06	<0.0001
SJC28	−4.59	<0.0001	−4.26	<0.0001	−6.11	<0.0001	−5.99	<0.0001
CDAI	5.87	<0.0001	−0.85	0.3949	−2.85	0.0044	−8.76	<0.0001

**C. SDAI**								
PGA	−3.56	0.0004	−1.77	0.0763	4.52	<0.0001	1.67	0.0955
MDGA	3.66	0.0003	9.13	<0.0001	13.58	<0.0001	6.79	<0.0001
TJC28	1.90	0.0575	0.06	0.9515	−0.31	0.7548	−2.37	0.0179
SJC28	−1.91	0.0555	−3.39	0.0007	−5.78	<0.0001	−3.52	0.0004
CRP	−1.54	0.1232	−2.4	0.0162	−7.27	<0.0001	−1.62	0.1050
SDAI	−0.86	0.3912	1.31	0.19	1.22	0.2233	−2.94	0.0033

*Visits, number of visits (%); PGA, patient assessment of disease activity (100 mm); TJC28, 28 tender joint count; SJC28, 28 swollen joint count; MDGA, physician assessment of disease activity (100 mm); CRP, C-reactive protein (mg/l); ESR, erythrocyte sedimentation rate (mm/h); DAS28-ESR, disease activity score evaluating 28 joints; CDAI, clinical disease activity index; SDAI, simplified disease activity index; *z, z*-test for equality of two independent means (*z* > 0 if the mean for the Dutch population was higher than that of the Portuguese)*.

In Dutch centers, SJC28 was positively correlated with TJC28 (*r* = 0.56; *p* < 0.001), with ESR (*r* = 0.23; *p* < 0.001) and with CRP (*r* = 0.32; *p* < 0.001). TJC28 was also positively correlated with ESR (*r* = 0.15; *p* < 0.001) and CRP (*r* = 0.17; *p* < 0.001). The same findings were found for the visits made in Portuguese centers, where SJC28 were positively correlated with TJC28 (*r* = 0.66; *p* < 0.001), ESR (*r* = 0.28; *p* < 0.001), and CRP (*r* = 0.25; *p* < 0.001). TJC28 was also positively correlated with ESR (*r* = 0.21; *p* < 0.001) and CRP (*r* = 0.19; *p* < 0.001). For both countries, these correlations are weak with the exception of the correlation between SJC28 and TJC28 where a moderate/good correlation was found.

The relative contribution of each individual component (independent of the weight of the variable, given by the standard calculation formula) was also determined. For the CDAI, we found that the contribution of global assessments of disease activity was decreased, and the contribution of joint counts was increased, in the high disease activity states compared to remission. We identified similar results with the SDAI; however, we also found that CRP (not included in the CDAI) had less of a contribution in high disease states compared to remission. With respect to the DAS28-ESR, the relative contribution of the individual components was very similar across populations and across all disease activity categories. Finally, the relative contribution of the PGA was higher for visits in which the CDAI and SDAI classified the patient as in remission than when remission was determined by DAS28-ESR. As such, higher PGA values were reported for visits in which patients were determined to be in remission by the DAS28-ESR than by the CDAI/SDAI, and less than 50% of “DAS28-ESR remission” visits were also in the CDAI or SDAI remission category.

To eliminate the effect of the number of visits per patient, we performed a sensitivity analysis where one visit was selected at random to analyze per patient. For this, we found that while the sample size was reduced, the results were generally unchanged (Tables 6 and 7).

**Table 6:** Patients’ characteristics and disease activity assessment using a randomly selected visit per patient.

	Dutch		Portuguese			
	*N*	Patient	*N*	Patient	*z*	*p*-Value
**Characteristics**						
Female (%)	5,870	69.5	3,185	82.1		<0.0001
	3,923		2,616			
Age (years) (mean ± SD)	5,489	62.6 ± 14.7	3,180	61.7 ± 14.2		0.003
Disease duration (years) (mean ± SD)	2,278	11.8 ± 8.9	2,305	15.0 ± 10.1		<0.0001
Diagnosis duration (years) (mean ± SD)	2,520	10.4 ± 8.7	2,397	12.8 ± 9.0		<0.0001

**Disease activity assessment**						
PGA (mean ± SD)	4,953	35.1 ± 23.7	2,134	38.5 ± 25.5		<0.0001
MDGA (mean ± SD)	1,939	28.3 ± 22.0	1,472	26.6 ± 21.1		0.023
TJC28 (mean ± SD)	5,327	2.6 ± 4.2	2,509	3.9 ± 5.8		<0.0001
SJC28 (mean ± SD)	5,272	1.6 ± 2.9	2,509	2.2 ± 3.7		<0.0001
ESR (mean ± SD)	4,874	18.9 ± 17.4	2,364	26.9 ± 22.7		<0.0001
CRP (mg/l) (mean ± SD)	961	9.2 ± 15.3	2,212	12.5 ± 22.7		<0.0001

**Disease activity scores**						
DAS28 Remission (%)	1,804	39.68%	564	27.78%	5.38	<0.0001
DAS28 Low (%)	847	18.63%	310	15.27%	1.38	0.1688
DAS28 moderate (%)	1,522	33.48%	790	38.92%	−2.57	0.0102
DAS28 high (%)	373	8.21%	366	18.03%	−3.99	0.0001
CDAI remission (%)	332	18.15%	205	14.25%	1.21	0.2266
CDAI Low (%)	721	39.42%	555	38.57%	0.31	0.757
CDAI moderate (%)	538	29.41%	439	30.51%	−0.37	0.711
CDAI high (%)	238	13.01%	240	16.68%	−1.13	0.259
SDAI remission (%)	70	13.21%	190	14.21%	−0.21	0.8335
SDAI low (%)	157	29.62%	510	38.15%	−2.01	0.044
SDAI Moderate (%)	205	38.68%	440	32.91%	1.42	0.1566
SDAI high (%)	98	18.49%	197	14.73%	−3.96	0.0001
ACR/European League Against Rheumatism Remission (%)	77	10.03%	219	11.43%	−1.08	0.282

*PGA, patient assessment of disease activity (100 mm); MDGA, Physician assessment of disease activity (100 mm); TJC28, 28 tender joint count; SJC28, 28 swollen joint count; ESR, erythrocyte sedimentation rate (mm/h); CRP, C-reactive protein (mg/l); DAS28, disease activity score evaluating 28 joints; CDAI, clinical disease activity index; SDAI, simplified disease activity index*.

*Chi-square and t-tests were used as appropriate. Significant p-value < 0.05*.

**Table 7:** Comparison of the mean of the components of the disease activity indices within each disease activity state between Dutch and Portuguese populations using one randomly selected visit per patient.

	Remission		Low		Moderate		High	
	*z*	*p*-Value	*z*	*p*-Value	*z*	*p*-Value	*z*	*p*-Value
**A. DAS28-ESR**								
PGA	1.26	0.2079	0.51	0.6096	3.29	0.001	3.29	0.001
TJC28	4.1	<0.0001	3.33	0.0009	−0.39	0.6988	−3.08	0.0021
SJC28	1.68	0.0932	1.58	0.1151	1.65	0.099	0.49	0.6267
ESR	−5.85	<0.0001	−3.54	0.0004	−5.65	<0.0001	−2.26	0.0241
DAS28-ESR	−3.36	0.0008	−0.03	0.9799	−2.73	0.0064	−2.09	0.0364
**B. CDAI**								
PGA	−0.51	0.6132	−3.64	0.0003	2.18	0.0296	1.31	0.19
MDGA	3.75	0.0002	4.76	<0.0001	6.57	<0.0001	3.77	0.0002
TJC28	0.94	0.3449	−0.37	0.7124	−4.46	<0.0001	−3.93	0.0001
SJC28	−2.58	0.0098	−0.69	0.4895	−1.74	0.0811	−1.21	0.2262
CDAI	0.96	0.339	−1.02	0.3081	−0.54	0.5865	−2.56	0.0105
**C. SDAI**								
PGA	−2.37	0.0178	−2.73	0.0062	1.27	0.2042	0.59	0.5526
MDGA	1.79	0.0739	5.62	<0.0001	9.13	<0.0001	3.1	0.0019
TJC28	0.93	0.3516	1.38	0.1677	0.24	0.808	−0.05	0.9585
SJC28	−0.97	0.3312	0.1	0.9224	−0.6	0.5506	0.26	0.7943
CRP	−1.42	0.157	−2.27	0.0234	−4.62	<0.0001	−2.8	0.0051
SDAI	−1.14	0.2532	1.03	0.3012	2.4	0.0166	−0.23	0.8171

*Visits, number of visits (%); PGA, patient assessment of disease activity (100 mm); TJC28, 28 tender joint count; SJC28, 28 swollen joint count; MDGA, physician assessment of disease activity (100 mm); CRP, C-reactive protein (mg/l); ESR, erythrocyte sedimentation rate (mm/h); DAS28-ESR, disease activity score evaluating 28 joints; CDAI, clinical disease activity index; SDAI, simplified disease activity index; *z, z*-test for equality of two independent means (*z* > 0 if the mean for Dutch group is higher than for Portuguese group)*.

## Discussion

Here, we have compared the DAS28-ESR, CDAI and SDAI indices in clinical practice in both Portuguese and Dutch populations using a large sample of RA patients retrieved from the multinational METEOR database. These populations had different sociodemographic characteristics (the Portuguese population included more women and had a longer disease duration); however, our primary aim was to compare the performance of the three indices and their individual components, not the disease activity within each population. Nevertheless, we found differences in disease activity between Portuguese and Dutch populations, with the exception for MDGA values.

We observed that use of the DAS28-ESR to characterize patients’ disease activity states resulted in larger differences in the proportion of patients assigned to each state between the Dutch and Portuguese populations, compared to the CDAI and SDAI assessments. We also found that ESR which is weighted more substantially in the DAS28-ESR than the other evaluations, differed the most between the two populations, which might explain the level of disagreement observed between the Dutch and Portuguese populations when using this assessment (15). The higher levels of ESR in RA patients seen in Portuguese centers may be explained by the higher prevalence of obesity found in Portuguese population as compared to the Dutch (16). However, in our study, we did not have data to confirm this hypothesis. For the CDAI and SDAI, PGA was the most influential measure across all disease activity states, except for high disease activity. This could be due to the subjective nature of the PGA, as patients’ assessments of disease activity may vary depending on their individual characteristics, such as personality, sociodemographic factors, or culture (7, 17–20). Taken together, our results suggest that, by weighting the individual components of the assessment differently, the DAS28-ESR and the CDAI/SDAI may result in different classifications of RA disease activity.

In addition, there is an ongoing discussion about the appropriateness of cut-off values for therapy response criteria, as debated in a paper by González-Álvaro and colleagues (21). Remission assessed by both the CDAI and the SDAI were found to be more stringent than the DAS28-ESR score, but less so than the ACR/EULAR remission criteria. This is in agreement with previous findings (11, 22) and suggests that the DAS28-ESR value of 2.6 may not be the most appropriate remission cut-off point (23). Moreover, in our study, the mean PGA value for patients with a DAS28-ESR score of less 2.6 is approximately twice than the PGA value for patients classified in remission according to the ACR/EULAR Boolean criteria. The low impact that a high PGA has in the DAS28-ESR seemed to be the primary reason for the significantly higher percentage of DAS28-ESR visits classified as in remission in the Dutch clinics, compared to Portuguese. Conversely, other reports have investigated potential problems stemming from the inclusion of the PGA as a component of the ACR/EULAR remission criteria (24), and these same issues likely extend to the CDAI and SDAI, due to the heavy contribution of the PGA in these indices.

Another interesting observation of this study was that the Portuguese MDGA was lower than that of the Dutch, regardless of disease activity state, and was usually discordant from ESR and joint counts. This surprising result was also found in the Quantitative Standard Monitoring of Patients with RA (QUEST-RA) registry that included data from 30 countries, where significant intercenter variation for MDGA and ESR was present (25).

There are some limitations to our analysis that must be considered when interpreting our results. METEOR is a large multinational database that gathers information on daily clinical practice, allowing for comparisons across different RA populations. However, there were missing values for some variables, namely for CRP, patients’ therapies and MDGA (in particular in Dutch population), which limited our analysis. In addition, some relevant variables were not included in the METEOR database, such as comorbidities, body mass index and level of education. Therefore, we were unable to include an assessment of these factors.

Historically, disease activity states were defined based on the physician’s decision and the drugs that were prescribed (26). However, now physicians rely on evaluations of disease activity states to decide the appropriate treatment and management strategies (27). Therefore, an understanding of the disease activity indices used in clinical practice is critical and may result in novel insights that can be used to develop new evaluations and/or improve the existing composite indices, such as by establishing new disease activity cutoffs that may be more appropriate for different populations. Here, we report that the DAS28-ESR and the CDAI/SDAI weight their components differently, sometimes resulting in discordant assessments of RA disease activity. Therefore, these indices should not be used interchangeably. In addition, these tests incorporate subjective assessments as well as objective measurement, such as PROs and MDGA, which may be influenced by cultural and educational backgrounds. Moreover, a physician’s evaluation may differ from the patient’s perspective and, as was observed in this study, may also differ from objective assessments such as swollen joint counts. Since the concept of “treat-to-target” depends on well-defined targets, it is necessary to revise the targets in RA and achieve a standardized and consistent evaluation method before this concept can be applied successfully.

## Conclusion

Here, we used the METEOR multinational database to analyze data from a total of 24,605 RA visits to Dutch and Portuguese clinics. We compared the outcomes of three methods to assess RA disease activity states; specifically, the DAS28-ESR, the CDAI, and the SDAI. We found that the percentage of Dutch and Portuguese visits classified as “in remission” was very similar when using the CDAI, the SDAI and the ACR/EULAR remission criteria. However, use of the DAS28-ESR resulted in a significantly higher proportion of remission classifications at Dutch clinics. In addition, we found that Portuguese physicians tended to classify patients into lower disease activity states than Dutch physicians.

Taken together, our results indicate that the DAS28-ESR and the CDAI/SDAI weights their individual components differently, which sometimes caused discordant assessments of RA disease activity. Based on our findings, a more consistent and standardized approach for classifying RA disease activity may be necessary, and the evaluations used may need to be adapted to better suit differences between individual populations.

## Ethics Statement

Reuma.pt was approved by Portuguese National Data Protection Board (CNDP) and participant hospitals Ethics Committee. METEOR was approved by local Ethics Committees. All study procedures were in accordance with Declaration of Helsinki.

## Author Contributions

HC, AR, JG, MS, JF, and JS contributed to the conception, design and organization of the study and critique of the manuscript. HC, AR, MG, SD, JG, MS, AF, JC, CA, EG, DH, PM, JB, JF, and JS contributed to the acquisition of data and/or the statistical analyses. HC, AR, MG, JG, MS, EG, PM, JF, and JS contributed to data interpretation and drafted the manuscript. All authors reviewed and accepted the final version of the manuscript.

## Conflict of Interest Statement

The authors declare that there are no conflicts of interest regarding the publication of this paper.
